# Ciprofloxacin-Resistant *Shigella sonnei* Associated with Travel to India

**DOI:** 10.3201/eid2105.141184

**Published:** 2015-05

**Authors:** Niall De Lappe, Jean O’Connor, Patricia Garvey, Paul McKeown, Martin Cormican

**Affiliations:** University Hospital Galway, Galway, Ireland (N. De Lappe, J. O’Connor, M. Cormican);; Health Protection Surveillance Centre, Dublin, Ireland (P. Garvey, P. McKeown)

**Keywords:** *Shigella sonnei*, bacteria, ciprofloxacin, antimicrobial drug resistance, travel, India

**To the Editor:** Shigellosis is an uncommon infection in many industrialized countries, and many cases are linked to travel to *Shigella* spp.–endemic countries. The epidemiology of *Shigella* infections in developing countries is changing. *S. sonnei* seems to be replacing the more antigenically diverse *S. flexneri* in regions undergoing economic development and improvements in water quality (*1*).

In 2012, a total of 29 cases of shigellosis were reported in Ireland through the Computerized Infectious Disease Reporting system (crude incidence rate 0.63 cases/100,000 population). Isolates from 20 (69%) of those 29 cases were submitted to the National Reference Laboratory in Galway, Ireland, for additional typing. In 2013, a total of 43 isolates were submitted for typing, more than double the 20 isolates submitted for 2012. This increase may be associated with a change in diagnostic methods: the increasing use of molecular methods for primary testing (*2*). During 2010–2013, the most common isolates were *S. sonnei* (54%) and *S. flexneri* (38%).

Isolate identification was confirmed by using VITEK 2 (bioMérieux, Marcy l’Etoile, France) and serotyping performed by using slide agglutination with commercial antisera (Sifin, Dusseldorf, Germany, and Mast, Liverpool, UK). Antimicrobial drug susceptibility testing was performed with disk-diffusion tests or Etests (2000–2009) and by broth microdilution (2010–2013) (Sensititre, Trek Diagnostic Systems, Cleveland, OH, USA). Susceptibility to ampicillin, chloramphenicol, streptomycin, sulfamethoxazole, tetracycline, trimethoprim, naladixic acid, ciprofloxacin, gentamicin, ceftazidime, cefpodoxime, and cefotaxime was assessed by using criteria from the European Committee on Antimicrobial Susceptibility Testing (http://www.eucast.org/clinical_breakpoints). Since October 2013, testing has also included azithromycin, tigecycline, meropenem, and cefepime. Pulsed-field gel electrophoresis (PFGE) was performed on all *S. sonnei* isolates by using the PulseNet method developed by the Centers for Disease Control and Prevention (*3*). Fisher exact test was applied to assess the significance of the association of ciprofloxacin resistance with reported travel to the subcontinent of India.

Although infection with *S. sonnei* is generally self-limiting, antimicrobial drug therapy is necessary for some patients and may reduce duration of shedding in feces (*4*). Ciprofloxacin is widely recommended for use in the absence of susceptibility test results. Alternative agents for therapy include ceftriaxone and azithromycin.

For 2000–2009, none of the 65 *S. sonnei* isolates submitted for typing were resistant to ciprofloxacin. For 2010–2013, the number of ciprofloxacin-resistant *S. sonnei* isolates and the total number of *S. sonnei* isolates submitted for testing were 6/17 (2010), 2/20 (2011), 4/12 (2012), and 12/23 (2013). All 24 ciprofloxacin-resistant isolates were co-resistant to trimethoprim, and all but 2 were also resistant to streptomycin, sulfamethoxazole, and tetracycline. Cefotaxime resistance in 1 isolate was associated with extended-spectrum β-lactamase production. Azithromycin resistance has not been detected since testing for this resistance began in October 2013.

All 24 isolates had indistinguishable or closely related (>92%) *Xba*I-PFGE profiles (Figure). The *Xba*I cluster also included 21 of 50 ciprofloxacin-susceptible *S. sonnei* isolates submitted during 2000–2013. Use of a second enzyme (*Bln*I) on a subset of the 24 isolates confirmed the close relationship among these 24 isolates (data not shown).

**Figure:**
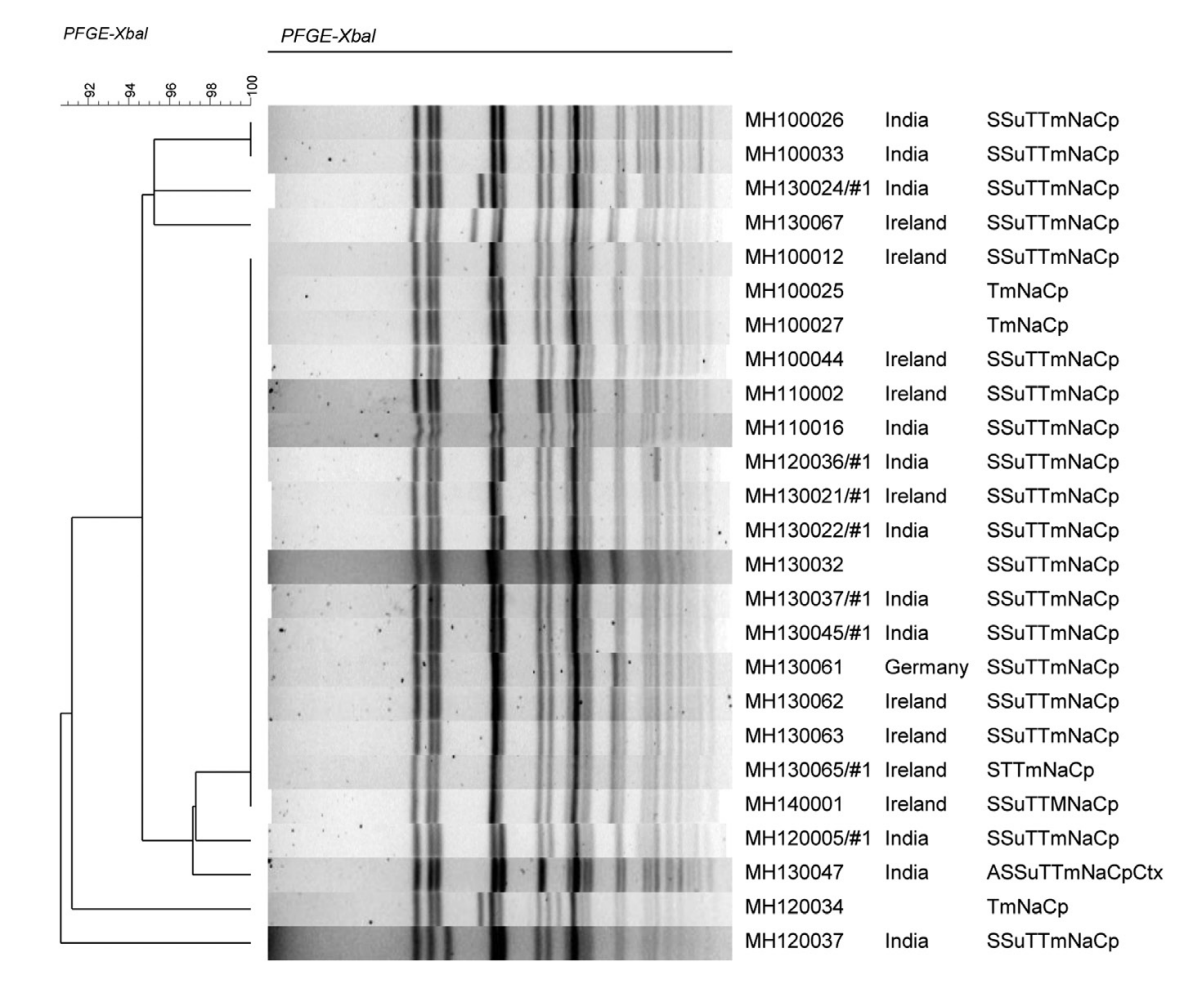
Dendrogram of ciprofloxacin-resistant *Shigella sonnei* digested with *Xba*I enzyme. Isolate identification numbers and country location for origin of infection are shown. In column on far right, antibiogram abbreviations indicate resistance to antimicrobial drugs: A, ampicillin; S, streptomycin; Su, sulfamethoxazole; T, tetracycline; Tm, trimethoprim; Na, nalidixic acid; Cp, ciprofloxacin; Ctx, cefotaxime. Scale bar indicates evolutionary distance. PFGE, pulsed-field gel electrophoresis.

Data from the Computerized Infectious Disease Reporting system (2010–2013) identified 72 reported cases of *S. sonnei* infection, of which 24 were ciprofloxacin resistant. Of 15 isolates associated with travel to the subcontinent of India, 11 were ciprofloxacin resistant, but of 47 other isolates for which the country of infection was reported, only 9 were ciprofloxacin resistant, a significant difference (p<0.0001).

International concern is growing regarding antimicrobial drug resistance in *Shigella* infections associated with India. Fluoroquinolone resistance emerged in *S. dysenteriae* in 2002, in *S. flexneri* in 2004, and in *S. sonnei* in 2007 (*5*). Studies from Japan have also reported an association between travel to India and infection with an *S. sonnei* clonal group that was multidrug resistant, including resistance to nalidixic acid (*6*). Furthermore, ciprofloxacin-resistant *S.* *sonnei* isolates from foodborne outbreaks in India in 2009 and 2010 (*7*) had *Xba*I- PFGE types and resistance profiles visually indistinguishable from those reported in our study. A study of *S. sonnei* isolates in Bhutan showed that this clonal group was also common there (*8*). Furthermore, a 2010 outbreak of ciprofloxacin-resistant *S. sonnei* in Canada associated with men who have sex with men showed *Xba*I- and *Bln*I-PFGE patterns that appear similar to the patterns for isolates in this study (*9*).

Antimicrobial drug resistance is a major global problem that is likely to be exacerbated in places with poor sanitation and intensive use of antimicrobial drugs in humans and animals. These factors have contributed to increased ciprofloxacin resistance in *Salmonella enterica* serovars Typhi and Paratyphi A (*10*).

A review of published literature and informal communication indicates that our observation of ciprofloxacin resistance in *S. sonnei* infections associated with travel to India is part of a general global trend. This increasing resistance suggests that ciprofloxacin may no longer be suitable for empiric therapy for *S. sonnei* infection, particularly for patients with a history of travel to the subcontinent of India.
